# Rapid Glucocorticoid-Induced Activation of TRP and CB1 Receptors Causes Biphasic Modulation of Glutamate Release in Gastric-Related Hypothalamic Preautonomic Neurons

**DOI:** 10.3389/fnins.2013.00003

**Published:** 2013-01-31

**Authors:** Carie R. Boychuk, Andrea Zsombok, Jeffrey G. Tasker, Bret N. Smith

**Affiliations:** ^1^Department of Physiology, University of Kentucky College of MedicineLexington, KY, USA; ^2^Department of Cell and Molecular Biology and Neuroscience Program, Tulane UniversityNew Orleans, LA, USA

**Keywords:** cannabinoid, vanilloid, paraventricular nucleus

## Abstract

Glucocorticoids rapidly regulate synaptic input to neuroendocrine cells in the hypothalamic paraventricular nucleus (PVN) by inducing the retrograde release of endogenous messengers. Here we investigated the rapid effects of dexamethasone (DEX) on excitatory synaptic input to feeding-related, preautonomic PVN neurons using whole-cell patch-clamp recordings. In ∼50% of identified gastric-related preautonomic PVN neurons, DEX elicited a biphasic synaptic response characterized by an initial rapid and transient increase in the frequency of miniature excitatory postsynaptic currents (mEPSCs), followed by a decrease in mEPSC frequency within 9 min; remaining cells displayed only a decrease in mEPSC frequency. The late-phase decrease in mEPSC frequency was mimicked by the cannabinoid receptor agonists anandamide (AEA) and WIN 55,212-2, and it was blocked by the CB1 receptor antagonist AM251. The biphasic DEX effect was mimicked by AEA. The early increase in mEPSCs was mimicked by activation of transient receptor potential vanilloid type 1 (TRPV1) receptors with capsaicin and by activation of TRPV4 receptors with 4-α-PDD. The increase was reduced, but not blocked, by selective TRPV1 antagonists and in TRPV1 knockout mice; it was blocked completely by the broad-spectrum TRPV antagonist ruthenium red and by combined application of selective TRPV1 and TRPV4 antagonists. The DEX effects were prevented entirely by intracellular infusion of the G-protein inhibitor, GDPβS. Thus, DEX biphasically modulates synaptic glutamate onto a subset of gastric-related PVN neurons, which is likely mediated by induction of a retrograde messenger. The effect includes a TRPV1/4 receptor-mediated transient increase and subsequent CB1 receptor-mediated suppression of glutamate release. Multiphasic modulation of glutamate input to PVN neurons represents a previously unappreciated complexity of control of autonomic output by glucocorticoids and endogenous cannabinoids.

## Introduction

The paraventricular nucleus (PVN) of the hypothalamus plays a critical role in integrating neural and humoral signals leading to the coordination of neuroendocrine and autonomic motor outputs. Preautonomic PVN neurons control sympathetic and parasympathetic output via projections to the brainstem and spinal cord (Swanson and Kuypers, 1980). Anatomical and physiological evidence suggests that PVN neurons are involved in controlling energy homeostasis, including regulation of feeding behavior and digestion (Sims and Lorden, 1986). In general, inhibition of PVN neuron activity tends to induce feeding, whereas a reduction in feeding is associated with increased PVN activity (Kow and Pfaff, 1989). This is presumably mediated in large part by neural interactions with brainstem autonomic nuclei that regulate output to the stomach. Modulation of hypothalamic-brainstem circuit activity likely plays a key role in regulating feeding behavior.

Glucocorticoids can affect the hypothalamic-pituitary-adrenal (HPA) axis and also regulate feeding behavior (Tempel et al., 1993; Dallman, 2003; Di et al., 2003). Recent findings indicate that glucocorticoids elicit a rapid inhibitory response in the PVN by inducing retrograde release of the endogenous cannabinoids (eCBs) 2-arachidonoyl glycerol (2-AG) and anandamide (AEA) from parvocellular and magnocellular neuroendocrine cells (Di et al., 2003, 2005b). These eCBs act to inhibit glutamate release, leading to a reduced excitatory synaptic drive to PVN neuroendocrine cells over the course of a few minutes (Di et al., 2005b). Cannabinoid actions in the brain are largely mediated through CB1 receptors, but AEA also activates TRPV1 in transfected cells (Zygmunt et al., 1999; Van Der Stelt and Di Marzo, 2004). Whereas CB1 receptors are G-protein-coupled receptors and their binding typically inhibits synaptic release, TRPV1 are ligand-coupled non-specific cation channels, whose activation at synaptic terminals typically increases transmitter release (Marinelli et al., 2003; Tominaga and Tominaga, 2005; Caterina, 2007). We recently identified dual inhibitory and excitatory effects of exogenously applied AEA on synaptic input to preautonomic neurons in the dorsal motor nucleus of the vagus, an effect that was mediated by binding to CB1R and TRPV1, respectively (Derbenev et al., 2004, 2006). TRPV1 receptors are expressed in the PVN (Cristino et al., 2006), and their activation appears to potentiate glutamatergic signals to PVN neurons that project to the spinal cord (Li et al., 2004). Recently, TRPV2 and TRPV4 were also identified in the hypothalamus (Guler et al., 2002; Wainwright et al., 2004; Cristino et al., 2006). Like TRPV1, TRPV4 can also be modulated by eCBs (Guler et al., 2002; Watanabe et al., 2003; Wechselberger et al., 2006). However, TRPV-mediated effects of eCBs have not previously been identified in PVN neurons.

Glucocorticoids applied in the PVN cause the release of eCB ligands from neuroendocrine PVN cells (Di et al., 2005b; Evanson et al., 2010). Since both CB1R and TRPV1 are functional in the PVN, and AEA is an agonist at both receptors and at TRPV4, we hypothesized that glucocorticoid-mediated eCB release activates both TRPV and CB1 receptors on presynaptic afferent terminals in PVN. Because of the known link between eCB activity and ingestive/digestive behaviors, we tested for DEX-induced modulation of miniature excitatory postsynaptic currents (mEPSCs) in gastric-related preautonomic PVN neurons identified by retrograde transsynaptic labeling from the stomach with pseudorabies virus (PRV) 152 (PRV-152), which expresses enhanced green fluorescent protein (EGFP).

## Materials and Methods

### Animals

Male (4–8 weeks old) Sprague Dawley rats (Harlan, Indianapolis, IN, USA) and female and male B6.129 × 1-Trpv1^tm1Jul^/J homozygote mice (6–19 weeks old a gift of Dr. Y. Lee, bred from founders obtained from The Jackson Laboratory, Bar Harbor, ME, USA) were used for these experiments. Animals were housed in a vivarium under 12 h light, 12 h dark cycle with food and water available *ad libitum*. The University of Kentucky Animal Care and Use Committee approved all animal procedures.

### Retrograde transsynaptic neuronal tracing

A PRV strain isogenic with the attenuated PRV Bartha, which was constructed to report EGFP (PRV-152; a gift from L. W. Enquist, Princeton University) was used to retrogradely label neurons that were synaptically connected to the gastric musculature (Smith et al., 2000; Davis et al., 2003; Glatzer et al., 2003; Derbenev et al., 2004, 2006). Under isoflurane anesthesia, rats were inoculated with PRV-152 (1 × 10^8^ pfu/ml) directed tangentially into the musculature of the greater curvature of the stomach fundus (2 μL; 3–4 injections per stomach) using a Hamilton syringe fitted with a 26-gage needle. Each injection was made over the course of 30 s and the needle remained in place for 1 min after injection. The abdominal compartment was rinsed thoroughly with sterile saline to prevent accidental labeling of other viscera (Glatzer et al., 2003; Derbenev et al., 2004, 2006). The abdominal wall was then sutured; animals recovered and were maintained in a biosafety level 2 laboratory for up to 96 h post-injection. The PRV-152 infects terminal fields in the stomach wall and selectively labels preautonomic central neurons retrogradely in synaptic series. Because it does not sort in axons or glial cells, the construct does not spread orthogradely or non-specifically from axons to other structures (Ch’ng et al., 2007). Labeling sequence, timing, and other characteristics have been well-characterized for this pathway and include initial appearance of EGFP in the PVN at about 72 h post-inoculation, with numbers of labeled cells peaking by 96 h (i.e., <24 h after initial neuron infection in the PVN). Central neurons infected for up to 72 h display no apparent adverse effects of viral infection (Smith et al., 2000; Irnaten et al., 2001; Davis et al., 2003; Glatzer et al., 2003, 2007; Derbenev et al., 2004), but this was verified in the PVN, where typical measurements of membrane potential, input resistance, action potential generation, and presence of low-threshold spikes indicative of preautonomic PVN cells were obtained (Luther and Tasker, 2000; Stern, 2001; Luther et al., 2002).

### PVN slice preparation

Acute hypothalamic slices containing the PVN were prepared from infected male Sprague Dawley rats and TRPV1 knockout mice. Animals were deeply anesthetized with halothane inhalation (Sigma, St. Louis, MO, USA) and then decapitated. The brain was removed from the cranial cavity after cutting the optic nerves and immersed in an ice-cold (0–4°C) artificial cerebral spinal fluid (aCSF) bubbled with 95% O_2_–5% CO_2_. The composition of aCSF was (in mM): 124 NaCl, 3 KCl, 26 NaHCO_3_, 1.4 NaH_2_PO_4_, 11 glucose, 1.3 CaCl_2_, and 1.3 MgCl_2_; pH 7.3–7.4. The hypothalamus was blocked and glued onto a metal stage and 300 μm slices were cut with a vibratome. The slices were transferred to a holding chamber and incubated in oxygenated aCSF at room temperature for 1 h before being transferred to a recording chamber mounted on the fixed stage of an upright microscope (BX51WI; Olympus, Melville, NY, USA).

### Whole-cell recordings

Whole-cell patch-clamp recordings were performed under visual control on an upright, fixed stage microscope equipped with infrared illumination and differential interference contrast (IR-DIC) and epifluorescence illumination to identify EGFP-labeled cells (Olympus BX51WI). Epifluorescence was briefly used to target EGFP-labeled cells, at which time the light source was switched to IR-DIC to obtain the recording. Gastric-related preautonomic neurons were identified as EGFP-labeled as previously described (Smith et al., 2000; Glatzer et al., 2003) by visualization under fluorescence microscopy and were filled with biocytin during recordings for *post hoc* visualization with avidin-Texas Red.

For whole-cell patch-clamp recordings, electrodes (2–5 MΩ) were filled with a solution containing the following (in mM): 130 Cs^+^-gluconate, 1 NaCl, 5 EGTA, 10 HEPES, 1 MgCl_2_, 1 CaCl_2_, 3 CsOH, 2–3 Mg-ATP, and 0.2% biocytin, pH 7.3–7.4, adjusted with 5 M CsOH. For current-clamp recordings, K^+^-gluconate was used in the recording pipette. To test whether these responses depended on G-protein activity in the recorded cell, a subset of neurons was recorded using pipettes containing guanosine 5′[β-thio]diphosphate trilithium salt (GDPβS; 500 μM, Sigma-Aldrich, St. Louis, MO, USA). Excitatory postsynaptic currents (EPSCs) were examined at a holding potential of −60 mV. Electrophysiological signals were recorded using an Axoclamp 700B amplifier (Molecular Devices, Union city, CA, USA), low-pass filtered at 2 kHz, and stored to a computer using a digidata 1440A A-D converter and pClamp 10.2 software (Molecular Devices, Union City, CA, USA). Synaptic currents were analyzed offline using pClamp 10.2 and MiniAnalysis 6.0.3. (Synaptosoft, Decatur, CA, USA).

### Drug application

All experiments were performed with tetrodotoxin (TTX, 1 μM) in the aCSF solution to block action potentials and isolate mEPSCs, with the exception of on-cell recordings of firing activity. Dexamethasone (DEX, 10 μM; Sigma, St. Louis, MO, USA) and ruthenium red (RR, 1 μM; Tocris, Ellisville, MO, USA) were dissolved in aCSF; capsaicin (CP, 0.1, 1, 10 μM), capsazepine (CPZ, 10 μM), 5′-iodoresiniferatoxin (5′-iRFT, 1 μM; Tocris Bioscience, Ellisville, MO, USA), and 4α-phorbol 12,13-didecanoate (4-α-PDD, 1 μM; Alexis Biochemicals, San Diego, CA, USA) were dissolved in ethanol and diluted in aCSF (final concentration of ethanol <0.01% by volume). WIN 55,212-2 (10 μM, Sigma), RN1743 (10 μM; Tocris), and AM251 (10 μM; Tocris) were dissolved in DMSO and diluted in aCSF. AEA (10 μM) was purchased as predissolved stock in Tocrisolve (Tocris). Vehicle alone had no effect on membrane or synaptic properties (Derbenev et al., 2006).

### Data analysis

Baseline (control) mean frequency and amplitude of mEPSCs were obtained over a 3 min period just prior to drug application. Measurements of drug effects within a given recording were assessed from the average of the first 3 min after drug application (i.e., 0–3 min after the drug equilibrated in the recording chamber; i.e., 3 min time point) and during min 6–9 (i.e., 9 min time point) of drug application. The effect of agonists and antagonists on mEPSC frequency and amplitude were analyzed using a repeated-measures ANOVA comparing control to segments examined 0–3 and 6–9 min after agonist application. When appropriate, Tukey’s *post hoc* test was used to determine significance. The non-parametric, intra-assay Kolmogorov–Smirnov test (K–S test; >150 events per condition) was also used initially to objectively determine if a given cell demonstrated a change in mEPSC frequency or amplitude during the first 3 min of drug application. A probability of <0.02 was considered significant for K–S analyses. In the population in which it was detected, the magnitude of change was assessed using a paired one-tailed Student’s *t*-test, where indicated. All other comparisons of single-variable grouped data were performed using paired, two-tailed Student’s *t*-test. Probability values < 0.05 were considered significant for grouped data. Values are reported as the mean ± SEM.

## Results

### Identification and membrane properties of gastric-related PRV-152-labeled neurons

**Figure 1:**
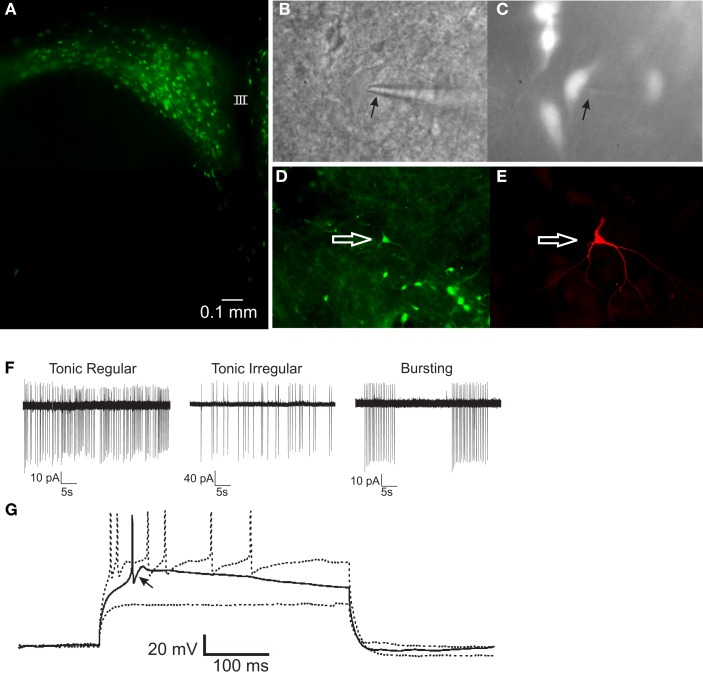
**PRV-152-infected gastric-related preautonomic neuron recorded in the PVN**. **(A)** Fluorescence micrograph of a PVN slice (300 μm) showing EGFP-labeled, gastric-related preautonomic neurons approximately 90 h after PRV-152 inoculation of the stomach. **(B)** Example of a PVN neuron that was recorded in the slice preparation. Arrow indicates to recording pipette. **(C)** The same neuron as in **(B)** illuminated with epifluorescence indicating it contained GFP. **(D)** Image of a slice from which a GFP-labeled PVN neuron was recorded. The arrow indicates the recorded cell. **(E)** Fluorescence image of the recorded cell [same as in **(D)**] filled with biocytin and visualized with an Avidin-Texas Red conjugate. **(F)** Loose-patch recordings of action potential activity demonstrating typical firing patterns in three PRV-152 labeled, gastric-related PVN neurons. **(G)** Current-clamp recording from a gastric-related preautonomic neuron that generated a low-threshold spike (arrow) in response to depolarizing current pulses (70–90 pA) from a membrane potential of −90 mV [same neuron as shown in **(D,E)**]. III, third ventricle.

Expression of EGFP in PVN neurons was observed beginning 72 h after PRV-152 inoculation of the stomach. Gastric-related preautonomic neurons labeled with EGFP were targeted for recording 72–96 h after inoculation (Figures 1A–E), a time window during which the electrophysiological properties of PRV-152-infected neurons were demonstrated previously to be unaffected by the virus (Smith et al., 2000; Glatzer et al., 2003, 2007). To confirm their identity as EGFP-labeled, gastric-related neurons and their location in PVN, a subset of recorded neurons was filled with biocytin and visualized *post hoc* with an avidin-Texas Red or avidin-rhodamine conjugate and/or an ABC-DAB reaction (Figure 1). Action potential firing and membrane properties of EGFP-labeled PVN neurons (Figures 1F,G) were examined and were found to be similar to those reported previously for PVN preautonomic neurons (Stern, 2001; Luther et al., 2002). Electrophysiological characteristics of gastric-related preautonomic PVN neurons included four basic firing patterns, including tonic regular, tonic irregular, bursting, and silent (Figure 1F, silent cells not presented), consistent with previous analyses of retrogradely labeled preautonomic and neuroendocrine PVN neurons (Stern, 2001; Luther et al., 2002). The presence of low-threshold spikes in response to depolarizing current pulses (70–90 pA) from a hyperpolarized membrane potential (−80 to −90 mV) was also observed, suggesting the activation of low-voltage activated calcium channels (Figure 1G).

### Biphasic effect of DEX on mEPSC frequency

**Figure 2:**
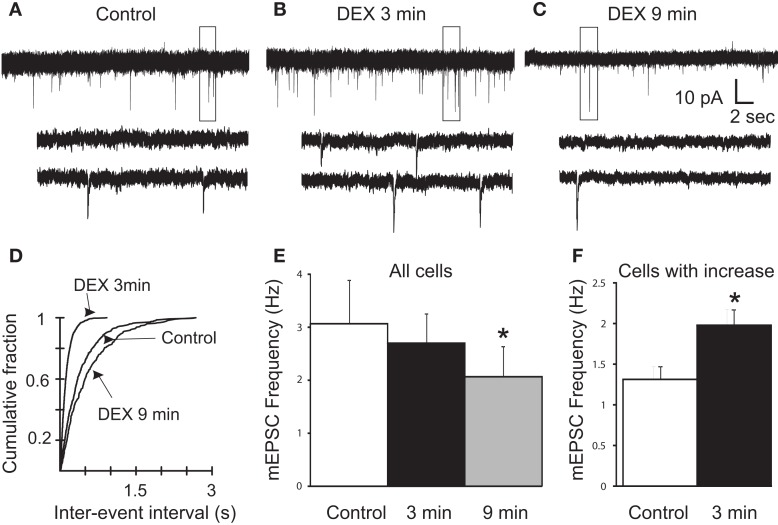
**Rapid biphasic changes in mEPSC frequency in PVN gastric-related preautonomic neurons elicited by dexamethasone (DEX)**. **(A–C)** Sequential recordings of mEPSCs observed in an EGFP-labeled PVN neuron at a holding potential of −60 mV in control conditions **(A)** and after 3 min **(B)** and 9 min **(C)** of bath application of 10 μM DEX. Bottom traces show the boxed areas in the upper traces on an expanded time scale. **(D)** Cumulative probability plots of inter-mEPSC interval distribution from the same cell showed a significant increase at 3 min (leftward shift) and a significant decrease at 9 min (rightward shift) in mEPSC frequency (K–S test, *p* < 0.02). **(E)** Mean group changes in mEPSC frequency caused by 10 μM DEX at 3 and 9 min (*n* = 11; ANOVA, Tukey’s; *p < 0.05 from control). **(F)** Mean group frequency changes in the subset of neurons in which the K–S test detected an increase in mEPSC frequency (*n* = 6; *p < 0.05, *t*-test).

Glucocorticoids were recently demonstrated to induce the retrograde release of eCBs from magnocellular and parvocellular neuroendocrine PVN neurons, leading to a presynaptic, CB1R-mediated reduction in mEPSC frequency within minutes of drug application (Di et al., 2003, 2005b). Here, mEPSCs were recorded to investigate the rapid effect of the synthetic glucocorticoid dexamethasone (DEX) on spike-independent glutamate release onto gastric-related preautonomic PVN neurons. Gastric-related preautonomic parvocellular PVN neurons demonstrated a significant decrease in mEPSC by 9 min of DEX application (Tukey’s; *p* = 0.0008; *n* = 11; Figure 2). Under control conditions, the average mEPSC frequency for all cells was 3.1 ± 0.8 Hz (*n* = 11) and ranged from 1.2 to 11.0 Hz. After 10 μM DEX application, the mean frequency of mEPSCs was decreased to 2.1 ±  0.6 Hz (range 0.7–7.4 Hz). The decrease in frequency at 9 min was detected in each of the 11 neurons. However, we also examined individual responses from 15 neurons during the first 3 min of DEX application and determined that 40% of the gastric-related preautonomic PVN neurons exhibited a significant increase in mEPSC frequency shortly after agonist application (K–S test; *p* < 0.02). Thus, in 6 out of 15 gastric-related preautonomic cells (40%), DEX-induced a rapid and transient increase in the mEPSC frequency within the first 3 min of drug exposure. Under control conditions, the average mEPSC frequency in these six neurons was 1.3 ± 0.2 Hz (range 0.9–1.8 Hz). The mean mEPSC frequency over the first 3 min DEX application (10 μM) increased to 2.0 ± 0.2 Hz (range 1.2–2.4 Hz; *p* < 0.05; paired *t*-test; *n* = 6; Figure 2). There was no correlation between the control mEPSC frequency in a given cell and the relative change in frequency subsequent to DEX application at either the 3 min (*R* = 0.0006) or 9 min (*R* = 0.0604) time points. There was no significant change in mEPSC amplitude during DEX application; the average amplitude before application of DEX was 11.5 ± 1 pA and after bath application of DEX was 10.4 ± 1 pA (*n* = 11; *p* > 0.05). Thus, whereas DEX application reduced mEPSC frequency in all neurons tested, this reduction was preceded by a transient enhancement in frequency in a large subset of neurons. In five neurons, mEPSC frequency was also examined 12–15 min after returning to normal ACSF to determine if the changes in mEPSC frequency were reversible. Return of mEPSC frequency to control levels 15 min after DEX application was apparent in only one cell, so recovery was not further examined.

### Biphasic effect of anandamide on mEPSC frequency

**Figure 3:**
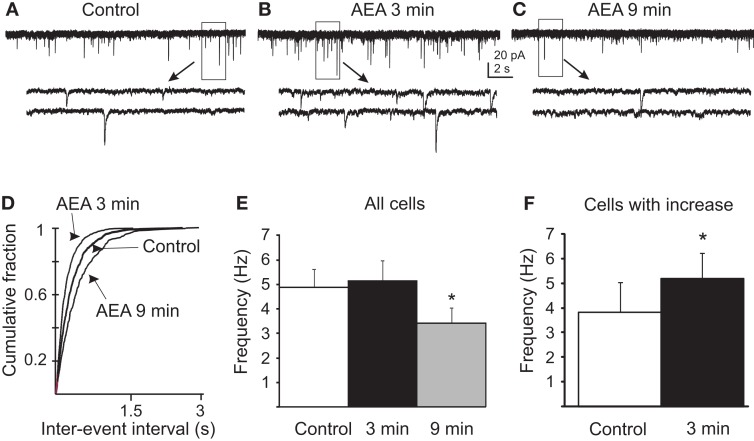
**Anandamide biphasically altered mEPSC frequency in gastric-related preautonomic neurons**. **(A–C)** Sequential recordings of mEPSCs observed in EGFP-labeled PVN neurons at a holding potential of −60 mV in control conditions **(A)** and after 3 min **(B)** and 9 min **(C)** of bath application of 10 μM AEA. Bottom traces show the boxed areas in the upper traces on an expanded time scale. **(D)** Cumulative probability plots of inter-mEPSC interval distribution from the same cell showed a significant increase at 3 min (leftward shift) and a significant decrease at 9 min (rightward shift) in mEPSC frequency (*p* < 0.02; K–S test). **(E)** Pooled data from all tested cells showing changes in frequency of mEPSCs caused by 10 μM AEA at 3 and 9 min (*n* = 9; *p < 0.05 from control and 3 min; ANOVA, Tukey’s). **(F)** Pooled data from the subset of neurons that responded to AEA with an increase in mEPSC frequency (45%) showing changes in the frequency of mEPSCs at 3 min (*n* = 5; * *p* < 0.05, *t*-test).

The effects of eCB agonists have been shown to involve cannabinoid (Di et al., 2003, 2005b) and/or vanilloid receptor activation (Derbenev et al., 2004, 2006) when these receptors are present centrally. Since the rapid glucocorticoid actions on glutamate release onto PVN neuroendocrine cells are mediated by eCB synthesis and retrograde release (Di et al., 2003, 2005b), we tested for the eCB-dependence of the biphasic DEX modulation of mEPSC frequency in gastric-related preautonomic PVN neurons. Similar to the DEX effect, bath application of 10 μM AEA biphasically altered mEPSC frequency (Figure 3). In the presence of AEA, all observed PVN cells demonstrated a decrease in mEPSC frequency by 9 min compared to both control and 3 min as determined by a Tukey’s *post hoc* (*n* = 9; *p* < 0.0001). The frequency of mEPSCs in control conditions for all neurons tested was 4.9 ± 0.7 Hz (range 1.5–8.1 Hz; *n* = 9). After a 9 min application of AEA (10 μM), the mEPSC frequency decreased to 3.4 ± 0.6 Hz (range 0.8–6.3 Hz; *p* < 0.001; Tukey’s; Figure 3E), which was similar to the inhibition of mEPSCs reported in PVN parvocellular and magnocellular neuroendocrine cells (Di et al., 2003, 2005b). Responses to AEA application within 11 individual cells revealed a subset of PVN neurons that demonstrated a rapid increase in mEPSC frequency (*p* < 0.02). The rapid, transient increase in mEPSC frequency was observed in 5 out of 11 gastric-related preautonomic cells (45%) in the first 3 min of the drug application (Figure 3). The average basal mEPSC frequency in these cells was 3.8 ± 1.2 Hz (range 0.8–7.6 Hz). After application of AEA (10 μM), the mean frequency of mEPSCs was increased to 5.2 ± 1.0 Hz (range 2.5–8.4 Hz; *n* = 5; *p* < 0.03; paired one-tailed *t*-test). There was no significant change in the mEPSC amplitude after application of AEA. The average amplitude before application of AEA was 14 ± 1 pA (range 9–19 pA; *n* = 11) and after 9 min application of AEA was 13.7 ± 1.2 pA (range 7–19 pA; *p* > 0.05). There was no correlation between the control mEPSC frequency in a given cell and the relative increase in frequency subsequent to AEA application at either the 3 (*R* = 0.1076) or 9 min (*R* = 0.1709) time points. The frequency of mEPSCs was therefore biphasically altered by AEA in a subset of neurons, but the overall effect was a decrease in mEPSC frequency by 9 min of AEA application.

### CB1 receptor dependence of DEX and AEA effect

**Figure 4:**
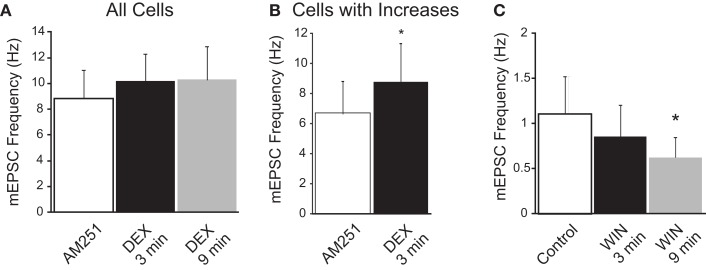
**CB1 receptor dependence of dexamethasone (DEX)-induced suppression of mEPSC frequency**. **(A)** In the presence of the CB1 receptor antagonist (AM251; 10 μM), DEX did not elicit a significant decrease in mEPSC frequency (*n* = 6; ANOVA, Tukey’s). **(B)** In AM251, DEX increased mEPSC frequency in a subset of neurons. The graph illustrates the mean frequency change in cells in which an increase in mEPSC frequency was detected (*n* = 4; *p < 0.05, *t*-test). **(C)** The synthetic cannabinoid agonist, WIN 55,212-2 (WIN; 10 μM) decreased the frequency of mEPSCs (*n* = 8; *p < 0.05 from control; ANOVA, Tukey’s); an increase in mEPSC frequency was not observed at either the early or late time point in the presence of WIN.

To determine if CB1 receptors participated in the DEX and AEA inhibition of mEPSC frequency, DEX and AEA were applied in the presence of the CB1R antagonist AM251 (10 μM). The mEPSC frequency was unaffected by AM251 alone, but the decrease in mEPSC frequency induced by DEX (10 μM) or AEA (10 μM) at 10 min was prevented by the antagonist. In the presence of AM251, a rapid increase in mEPSC frequency was observed after application of DEX or AEA. Bath application of 10 μM DEX in the presence of AM251 increased mEPSC frequency from 6.6 ± 2.1 Hz (range 2.9–12.2 Hz) to 8.7 ± 2.6 Hz (range 4.4–15.4 Hz; *p* < 0.05; paired *t*-test) in four out of seven cells during the first 3 min (57%; Figure 4B), while the mEPSC frequency in the remaining three cells was unaffected. However, no cell demonstrated a decrease in mEPSC frequency at either early or later time periods (Tukey’s, *p* = 0.12; Figure 4A). Similarly, bath application of 10 μM AEA in the presence of AM251 increased mEPSC frequency from 2.8 ± 0.6 Hz (range 2.0–4.1 Hz) to 4.3 ± 0.6 Hz (range 3.6–5.7 Hz; *p* < 0.02; paired *t*-test) in three out of seven cells (43%), but a decrease in mEPSC frequency was not detected during AEA application (Tukey’s, *p* = 0.58). Whereas the decrease in mEPSC frequency was blocked by the CB1 receptor antagonist, the transient increase in mEPSC frequency caused by DEX or AEA in a subset of neurons was not prevented.

The high-affinity synthetic cannabinoid agonist WIN 55,212-2 (WIN) decreased the mEPSC frequency in preautonomic parvocellular neurons. The K–S test did not detect a subset of neurons in which mEPSC frequency increased. Rather bath application of 10 μM WIN decreased mEPSC frequency in all cells tested measured by 9 min from 1.1 ± 0.3 Hz (range 0.4–3.4 Hz) to 0.6 ± 0.2 Hz (range 0.4–1.8 Hz; *p* < 0.04; Tukeys; *n* = 8; Figure 4C); there was no change in frequency in any recording at the earlier time point (*p* > 0.05). The inhibitory effect of DEX on mEPSC frequency was thus prevented by a CB1 receptor antagonist and mimicked by a CB receptor agonist. It was likely mediated, therefore, by the release of an eCB, similar to the effect described previously in PVN neuroendocrine cells (Di et al., 2003, 2005b).

### TRP receptor dependence of DEX and AEA effect

Anandamide is an agonist at TRPV1 (Di Marzo et al., 1994; Zygmunt et al., 1999) and effects of the TRPV1 agonist capsaicin have been reported previously in parvocellular PVN cells (Li et al., 2004). We hypothesized that the DEX- and AEA-induced transient increases in mEPSC frequency in preautonomic neurons were caused by the activation of TRPV receptors in the PVN. To determine if the rapid, transient increase in mEPSC frequency could be mimicked by a TRPV1 agonist other than AEA, the effect of capsaicin was investigated in PRV-152 labeled, gastric-related, preautonomic PVN neurons. Within 3 min of application, capsaicin increased the frequency of mEPSCs in EGFP-labeled preautonomic neurons. Bath application of 100 nM capsaicin increased mEPSC frequency in one neuron (i.e., by K–S test) but had no significant overall effect on the mEPSC frequency. Baseline frequency was 3.5 ± 0.9 Hz (range 0.8–4.9 Hz) and was 3.8 ± 1.0 Hz in 100 nM capsaicin (range 0.8–5.4 Hz; *p* > 0.05; two-tailed, paired *t*-test; *n* = 4). Application of 1 μM capsaicin increased significantly the frequency of mEPSCs in four of five neurons. Mean frequency was increased significantly in this group of cells, from 3.3 ± 1.0 Hz (range 1.3–6.5 Hz) to 5.2 ± 0.9 Hz (range 3.0–7.9 Hz; *p* < 0.05; *n* = 5). Application of 10 μM capsaicin increased mEPSC frequency in seven of eight neurons. Mean frequency was increased from 4.5 ± 1.1 Hz (range 0.8–7.7 Hz) to 8.3 ± 1.8 Hz (range 0.9–13.5 Hz; *p* < 0.05; *n* = 8). There was no correlation between the control mEPSC frequency in a given cell and the relative increase in frequency subsequent to capsaicin application (*R* = 0.1416). There was no significant change in mEPSC amplitude after application of any concentration of capsaicin (*p* > 0.05).

Application of the broad-spectrum TRPV antagonist ruthenium red (1 μM) blocked completely the rapid increase in mEPSC frequency caused by DEX (10 μM; *n* = 5), capsaicin (1 μM; *n* = 5) and AEA (10 μM, *n* = 8; Table 1) in gastric-related PVN neurons. In the presence of ruthenium red, DEX (10 μM) induced a 15 ± 6% decrease in mEPSC frequency instead of an increase at 3 min (compare to 53 ± 10% increase, Figure 2F), which then progressed to a 37 ± 6% decrease in mEPSC frequency by 9 min (*p* < 0.05; ANOVA, Tukey’s; *n* = 5); the decrease in mEPSC frequency at 9 min was similar to that seen in the absence of ruthenium red. Application of capsaicin (1 μM) was without effect in the presence of ruthenium red (*p* > 0.05; *n* = 5; Table 1). Ruthenium red prevented the rapid increase in mEPSC frequency by AEA in the first 3 min (Table 1), but did not affect the decrease in mEPSC frequency at 9 min (32 ± 9%; *p* < 0.05, *n* = 6). Ruthenium red (1 μM) alone had no effect on the baseline frequency of mEPSCs in any of four cells tested. It prevented the rapid, transient increase in mEPSC frequency caused by DEX, capsaicin, and AEA, whereas the later inhibition of mEPSCs by DEX and AEA was not blocked. These data implicated the involvement of a receptor from the TRPV family in the excitatory effect of DEX and AEA on mEPSC frequency (Table 1).

**Table 1:** **Percent of cells responding with an increase in mEPSC frequency and percent frequency change due to DEX, AEA, and capsaicin after TRPV receptor block**.

	DEX (10 μM)		AEA (10 μM)		Capsaicin (1 μM)	
	% Responding cells	% Frequency change	% Responding cells	% Frequency change	% Responding cells	% Frequency change
Control	40 (15)	53 ± 10	45 (11)	103 ± 72	80 (5)	113 ± 39
TRPV1 KO	–	–	–	–	57 (7)	36 ± 18
TRVP1 antagonist	40 (10)	32 ± 13	50 (10)	62 ± 34	64 (14)	92 ± 33
TRPV1 + TRPV4 antagonist	0 (5)	3 ± 4	–	–	–	–
Ruthenium red	0 (6)	−6 ± 4	0 (6)	−11 ± 2	0 (5)	−13 ± 13

*Numbers in parenthesis indicate total number of cells examined. AEA, anandamide; DEX, dexamethasone*.

To identify the possible receptor(s) mediating the rapid facilitatory effect of AEA, DEX, and capsaicin on mEPSC frequency, selective antagonists to TRPV1 were applied prior to application of capsaicin (1 μM), AEA (10 μM), and DEX (10 μM). A 15 min preapplication of neither the selective TRPV1 receptor antagonist capsazepine (10 μM) nor the potent TRPV1 antagonist 5′-iRFT (1 μM) blocked completely the excitatory effect of capsaicin, AEA, or DEX. The K–S test revealed a significant capsaicin-mediated increase in mEPSC frequency in four out of seven cells in the presence of 5′-iRFT (*p* < 0.05; Table 1). An increase in mEPSC frequency (62 ± 34%) induced by AEA (10 μM) was also seen in 5 of 10 cells in the presence of 5′-iRFT (*p* < 0.05). In addition, DEX (10 μM) increased mEPSC frequency (32 ± 13%) in 4 of 10 cells in the presence of capsazepine (10 μM, *p* < 0.05; K–S test; Table 1), while capsaicin (1 μM) increased mEPSC frequency in five of seven cells (Table 1). The percentage of neurons that responded to AEA and DEX application was similar in control conditions (∼50%) and in the presence of TRPV1 antagonist (∼50%), but the magnitude of the increase observed under control conditions (53 ± 10% for DEX; 102 ± 78% for AEA) was greater than that observed in the presence of the antagonist (32 ± 13% for DEX; 62 ± 34% for AEA), suggesting that a portion (∼50%) of the agonist activity was due to activation of TRPV1 (Table 1). In addition to capsaicin-induced TRPV1-mediated effects in PVN (Li et al., 2004), a non-TRPV1-mediated increase in mEPSC frequency was also elicited by capsaicin, AEA, and DEX when TRPV1 receptors were blocked.

### TRPV1 knockout mice

To verify that the capsaicin-mediated increase in mEPSC frequency was mediated by receptors other than TRPV1 and was not simply a result of agonists “outcompeting” antagonists, we determined the effect of capsaicin on mEPSCs in gastric-related PVN neurons in slices from TRPV1 knockout mice (B6.129 × 1-Trpv1^tm1Jul^/J homozygote). Absence of the nociceptive wiping response to ocular application of a capsaicin solution (200 μM) was used to verify the absence of TRPV1 functionality in the knockout mice (Gamse, 1982). Bath application of capsaicin (1 μM) significantly increased the frequency of mEPSCs in four out of seven cells from TRPV1 knockout mice within 3 min of application, from 2.9 ± 0.8 Hz (range 1.6–5.2 Hz) in control to 3.8 ± 0.8 Hz in capsaicin (range 2.4–5.8 Hz; *p* < 0.05; *n* = 4; Table 1).

**Figure 5:**
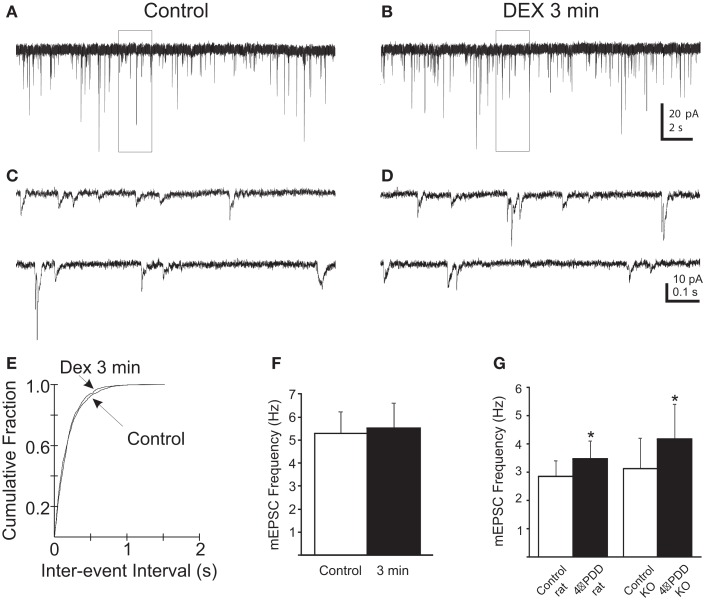
**Rapid increase in mEPSC frequency in PVN gastric-related preautonomic neurons elicited by dexamethasone (DEX) at 3 min depends on TRPV1 and TRPV4 receptors**. **(A,B)** Sequential recordings of mEPSCs observed in an EGFP-labeled PVN neuron at a holding potential of −60 mV in the presence of AM251 and the selective TRPV1 (5′-iRFT) and TRPV4 (RN1734) antagonists during control conditions **(A)** and after 3 min **(B)** of bath application of 10 μM DEX. **(C,D)** Expanded traces from the boxed areas in the traces shown in **(A,B)**. **(E)** Cumulative probability plots of inter-mEPSC interval distribution from the same cell showed no significant increase at 3 min in mEPSC frequency (*p* < 0.02; K–S test). **(F)** No change in group means in mEPSC frequency after by 10 μM DEX at 3 and 9 min (*n* = 5; *p* > 0.05; ANOVA, Tukey’s). **(G)** 4-α-PDD (1 μM), a selective TRPV4 receptor agonist, increased the mEPSC frequency in gastric-related preautonomic PVN neurons from rat (*n* = 4) and TRPV1 knockout mice (*n* = 6; *p* < 0.05; *t*-test).

Dexamethasone application resulted in a ruthenium red-sensitive enhancement of mEPSC frequency that was reduced, but was not blocked by selective TRPV1 antagonists. To determine if the DEX-induced changes in mEPSC frequency involved activation of TRPV4 in addition to TRPV1 and CB1 receptors, DEX was applied in the presence of a cocktail of AM251 (10 μM), 5′-iRFT (10 μM), and the TRPV4 antagonist RN 1734 (10 μM). In the presence of these combined selective antagonists, baseline mEPSC frequency was 5.3 ± 1.0 Hz (*n* = 5). DEX application failed to increase mEPSC frequency at 3 min (5.5 ± 1.1 Hz; *p* > 0.05) or decrease mEPSC frequency at 9 min (5.9 ± 1.2 Hz; *p* > 0.05; Table 1; Figure 5) in any neuron in the presence of the antagonists. Although a residual increase in mEPSC frequency was observed after TRPV1/CB1 receptor blockade alone, addition of a TRPV4 antagonist completely prevented the effects of DEX on mEPSC frequency.

### The effect of TRPV4 agonist

Recent evidence suggests that AEA may modulate TRPV4 activity in addition to TRPV1 and CB1R (Watanabe et al., 2003; Nilius et al., 2007). Because TRPV1 and TRPV4 have similar functional and pharmacological properties and are often co-expressed in the same brain regions (Cohen, 2007; Nilius et al., 2007), we examined TRPV4 activation by 4-α-PDD in gastric-related preautonomic PVN neurons of rats and TRPV1 knockout mice to verify the presence of functional TRPV4 receptors in these animals. In rats, bath application of 4-α-PDD (1 μM) increased mEPSC frequency in each of four cells, from 2.8 ± 0.5 Hz in control (range 1.6–4.3 Hz) to 3.5 ± 0.6 Hz in 4-α-PDD (range 2.2–5.1 Hz, *p* < 0.05, *n* = 4) at 3 min (Figure 5G). In TRPV1 knockout mice, bath application of 4-α-PDD (1 μM) increased mEPSC frequency in each of six neurons, from 3.1 ± 1 Hz (range 0.3–6.3 Hz) to 4.2 ± 1.3 Hz (range 0.6–8.3 Hz, *p* < 0.05; *n* = 6) by 3 min of drug application (Figure 5G). Like activation of TRPV1 receptors, activation of TRPV4 receptors also produced an increase in mEPSC frequency in gastric-related PVN neurons.

### Effect of DEX on mEPSC frequency depends on G-protein-mediated activity

**Figure 6:**
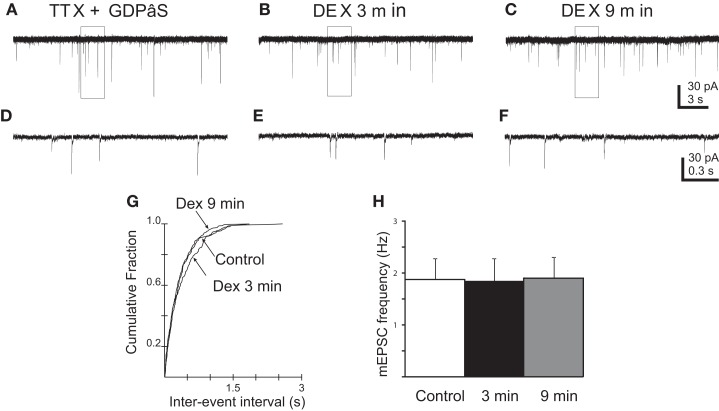
**Blockade of G-protein activity prevented DEX-induced changes in mEPSC frequency in PVN gastric-related preautonomic neurons**. **(A–C)** Sequential recordings of mEPSCs observed in an EGFP-labeled PVN neuron at a holding potential of −60 mV loaded with the G-protein inhibitor, GDPβS, during control conditions **(A)**, after 3 **(B)** and 9 min **(C)** of bath application of 10 μM DEX. **(D–F)** Expanded traces from the boxed areas in the traces shown in **(A–C)**. **(G)** Cumulative probability plots of inter-mEPSC interval distribution from the same cell showed no significant increase at any time in mEPSC frequency (*p* > 0.05; K–S test). **(H)** No change in group means in mEPSC frequency after by 10 μM DEX at 3 and 9 min (*n* = 5; *p* > 0.05; ANOVA, Tukey’s).

It was shown previously that DEX-induced eCB-mediated effects on synaptic input via a postsynaptic, G-protein-dependent effect in PVN neurons (Di et al., 2003, 2009). To determine if the effect of DEX on mEPSC frequency depended on G-protein-mediated activity in the recorded gastric-related neurons, cells were loaded intracellularly with GDP-β−S (500 μM) to inhibit G-protein function. The frequency of mEPSCs was 1.9 ± 0.4 Hz in five cells loaded with GDP-β−S (Figure 6). Application of DEX (10 μM) failed to alter mEPSC frequency in any neuron either during the first 3 min (1.8 ± 0.4 Hz; *p* > 0.05) or at 9 min (1.9 ± 0.4 Hz; *p* > 0.05), as determined by either the intra-assay K–S test or repeated-measures ANOVA. Blocking G-protein activity in recorded neurons with intracellular GDP-β−S, therefore, prevented the rapid excitatory and inhibitory effects of DEX.

## Discussion

In this study we present evidence for a novel rapid biphasic modulation by glucocorticoids and AEA of excitatory synaptic input to a subset of identified gastric-related preautonomic neurons in the PVN of the rat hypothalamus. This modulation occurs via dual activation of TRPV and CB1 receptors in a subset of neurons. To target the stomach-related preautonomic PVN neurons specifically, we used the transsynaptic retrograde transport of PRV-152, which expressed EGFP in pre-gastric neurons. Organ-specific labeling with PRV-152 after peripheral inoculation has been demonstrated previously (Larsen, 1999; Cano et al., 2000, 2001; Glatzer et al., 2003; Williams et al., 2007). Consistent with previous findings (Smith et al., 2000; Irnaten et al., 2001; Davis et al., 2003; Glatzer et al., 2003, 2007; Derbenev et al., 2004; Williams and Smith, 2006), we did not observe any obvious differences in the basic electrophysiological properties of PRV-152-labeled neurons versus those reported for preautonomic PVN neurons identified by other methods, including the presence of low-threshold spikes that characterize these neurons (Stern, 2001; Luther et al., 2002). Recorded PVN neurons expressing EGFP were, therefore, likely to be preautonomic cells with polysynaptic projections to the stomach.

### Activation of CB1 receptors

Previous data indicated that DEX activates a postsynaptic membrane-associated receptor in PVN neurosecretory neurons, which leads to the release of the eCBs, AEA, and 2-AG (Di et al., 2003, 2005a; Evanson et al., 2010). Immunohistochemical evidence indicates the presence of CB1R in the PVN (Cristino et al., 2006). We observed a significant inhibition of glutamate release in gastric-related preautonomic PVN neurons, as indicated by a decrease in mEPSC frequency by 9 min of DEX application. This was mimicked within a similar time frame by AEA and WIN 55,212-2 and was abolished by the CB1 receptor antagonist AM251. This finding is similar to observations in neuroendocrine parvocellular and magnocellular PVN cells, where glucocorticoids and cannabinoid agonists inhibited glutamatergic input to neuroendocrine cells (Di et al., 2003, 2005a). The inhibition of mEPSC frequency was likely mediated by CB1 receptor activation, as previously reported for neuroendocrine neurons of the PVN (Di et al., 2003, 2005b).

Whereas the enhancement of mEPSC frequency was observed in approximately half of gastric-related PVN neurons, the DEX- and AEA-induced decreases in mEPSC frequency were observed in all cells. The rapid enhancement of mEPSC frequency was not reported in neuroendocrine cells (Di et al., 2003, 2005b), which could indicate a functional difference in the response characteristics of these two cell groups. Interestingly, glucocorticoids were shown to elicit a facilitation of GABA release onto magnocellular neuroendocrine cells over several minutes, but this was mediated by retrograde nitric oxide actions and not by activation of presynaptic TRPV receptors (Di et al., 2005b, 2009). Thus, PVN neuron activity would be expected to be inhibited over several minutes of exposure to glucocorticoids, via suppression of glutamate release and enhancement of GABA release.

### Activation of TRPV receptors

We also observed a significant transient increase in mEPSC frequency within the first few min of DEX and AEA application in about 50% of gastric-related preautonomic PVN neurons, which was mimicked by capsaicin and the TRPV4-selective agonist 4-α-PDD and was therefore most likely due to activation of TRPV receptors. The present results are consistent with the hypothesis that both TRPV1 and TRPV4 are functional in the nucleus. Functional TRPV1 receptors have been reported in the PVN previously (Li et al., 2004), and it is possible that glucocorticoid-mediated release of AEA represents an endogenous means of activating these receptors (Smart et al., 2000). The initial excitatory phase of the biphasic response was blocked by the non-selective TRPV antagonist ruthenium red and was eliminated in the presence of combined antagonists to TRPV1 and TRPV4 receptors. However, selective antagonism of TRPV1 only partially blocked the effect, consistent with previous reports (Wenger et al., 2003; Karlsson et al., 2005). Glutamate release was also enhanced by the TRPV4 agonist 4-α-PDD, suggesting that TRPV4 receptors contribute to the facilitatory response to DEX and AEA (Vriens et al., 2004). Indeed, TRPV1 receptors are present in the PVN at presynaptic glutamatergic terminals (Mezey et al., 2000; Li et al., 2004; Cristino et al., 2006), and TRPV4 receptors have been identified in the hypothalamus (Guler et al., 2002; Carreno et al., 2009). In a subset of gastric-related circuits, therefore, AEA or DEX act first to activate presynaptically located TRPV receptors and enhance glutamate release, and subsequently to activate CB1 receptors and suppress glutamate release. In an extension of previous findings (Di et al., 2005b) that also showed glucocorticoid effects on synaptic release to be prevented by blocking G-protein-mediated responses in the recorded neuron, our data are consistent with a model in which an eCB or other retrograde messenger is released by rapid, G-protein-mediated glucocorticoid actions on gastric-related preautonomic neurons in the PVN.

The TRPV receptors (TRPV1-6) are a family of ligand-gated, non-selective cation channels with high Ca^2+^ permeability (Montell, 2001). They are activated by a wide range of stimuli (Nilius et al., 2003), including thermal, mechanical, and osmotic stimuli and endogenous lipids, including AEA, NADA, and arachidonic acid metabolites (Watanabe et al., 2003). Capsaicin has been demonstrated to significantly increase the frequency of mEPSCs (but not mIPSCs) without changing mEPSC amplitude or decay time constant in presympathetic PVN neurons (Li et al., 2004). These data are consistent with another study showing that capsaicin stimulates glutamate release from hypothalamic slices (Sasamura et al., 1998). A presynaptic effect of capsaicin on neurotransmitter release was also shown in feeding-related autonomic brainstem areas (Jin et al., 2004; Derbenev et al., 2006). Li et al. (2004) reported that selective TRPV1 blockade prevented the averaged effect of capsaicin, but did not report effects on individual neurons. A recent report indicated that TRPV1-mediated responses were restricted to liver-related preautonomic PVN neurons, and that this effect was eliminated in a model of type 1 diabetes (Gao et al., 2012) due to receptor internalization. This mechanism of diabetes-induced TRPV1 plasticity was previously identified in gastric-related preganglionic parasympathetic motor neurons of the dorsal motor nucleus of the vagus (Zsombok et al., 2011). Our data indicating that TRPV1 activates inputs to gastric-related PVN cells, in addition to liver-related cells described previously (Gao et al., 2012), suggest that TRPV1 modulation of glutamate release is not limited to cells regulating a specific organ system. It is also possible that individual PVN neurons regulate more than one digestive visceral organ via the vagus nerve, possibly via circuits within the vagal complex. However, expression of DEX-induced, TRPV-mediated excitation of glutamate release was not ubiquitous, occurring in approximately 40–50% of the pre-gastric neurons we recorded, which suggests that only a subpopulation of pre-gastric neurons within the PVN contain glucocorticoid-induced TRPV receptor activity.

We also found that the effect of capsaicin persisted in null mutant mice lacking TRPV1, consistent with the residual effect in the presence of TRPV1 antagonists. In these mice, and in rats, a selective TRPV4 agonist increased mEPSC frequency. The concept of both TRPV1 and TRPV4 operating in concert is not surprising, since recent work suggests an involvement of TRPV1 and/or TRPV4 in osmoregulation, where a possible interaction occurs between the receptors to mediate osmosensitivity (Sharif Naeini et al., 2006; Cohen, 2007). Hypotonicity activates TRPV4 (Watanabe et al., 2003; Vriens et al., 2004); hypertonicity activates TRPV1, or possibly a novel TRPV1 splice variant, found in the lamina terminalis and in magnocellular neurons of the PVN, which is present in TRPV1^-/-^ mice (Schumacher et al., 2000; Ciura and Bourque, 2006; Sharif Naeini et al., 2006; Tian et al., 2006). Our findings suggest a possible cellular mechanism by which the TRPV receptor family (i.e., TRPV1, TRPV4) operate in concert to maintain homeostatic autonomic functions in addition to osmoregulation.

In addition to being eCBs, AEA, and NADA are functional agonists at TRPV1 (Di Marzo et al., 1994; Zygmunt et al., 1999; Smart et al., 2000). TRPV1-mediated effects of the “endocannabinoids” AEA and NADA have been described in the brainstem (Derbenev et al., 2006; Sharkey et al., 2007) and the striatum (Maccarrone et al., 2008). These results demonstrated a functional role for TRPV1-mediated effects of eicosanoid-derived endovanilloids/endocannabinoids in the brain, similar to pharmacological actions in transfected cells (van der Stelt et al., 2005). Additionally, AEA can modulate TRPV4 receptor activity indirectly due to enzymatic hydrolysis to arachidonic acid and metabolism of arachidonic acid by cytochrome p450 (Watanabe et al., 2003), further supporting its role as an endogenous vanilloid. Our results are consistent with the hypothesis that endocannabinoids/endovanilloids, after either exogenous agonist application or endogenously induced release by DEX, can activate CB1, TRPV1, and TRPV4 receptors in the PVN.

### Possible physiological relevance

One possible functional role for the biphasic action of DEX in a subset of preautonomic neurons is to regulate the response bandwidth of these cells during transitions from low to high glucocorticoid levels. In neurons that are relatively quiescent prior to glucocorticoid exposure, a reduction in excitatory input would not be expected to have large effects. However, activation of TRPV receptors may “prime” the system for the eventual reduction in activity caused by CB1 receptor-mediated reduction in excitation and subsequent orexigenic activity. Alternatively, specific synapses may be differentially regulated by each receptor type. In either case, rapid and transient responses in centrally projecting neurons, whose activity is relayed to the next neuron in a synaptic chain with high fidelity, may allow for immediate changes in gastric modulation. Release of eCBs is thought to be “on demand” and to correspond to increases in cellular activity (Di Marzo et al., 1994). The transient increase in excitatory synaptic activity caused by eCB release may result in an increase in the gain of the system, such that the eventual inhibition of input becomes more relevant to the cell than it would normally be in the absence of the priming influence of TRPV activation. The broad regulation of neuroendocrine PVN neurons by DEX and AEA (Di et al., 2003, 2005b, 2009; Di and Tasker, 2008), which we also now report in preautonomic neurons, suggests a role for glucocorticoids in coordinating both endocrine and autonomic output from the nucleus. A biphasic regulation of gastric-associated outputs also provides an increased capacity for modulation of these circuits, as well as for plastic changes associated with different feeding-related states.

The present findings in identified gastric-related preautonomic PVN neurons support the hypothesis that feeding and digestive behaviors are rapidly modulated by the activity of glucocorticoids and AEA in the PVN and that this modulation is often biphasic. Typically, excitation of PVN neurons is associated with reduced feeding, while inhibition (or reduced excitation) in the PVN enhances feeding (Grandison and Guidotti, 1977; Sims and Lorden, 1986; Kow and Pfaff, 1989). The transient excitatory effect of eCB release would likely suppress gastric activity whereas the delayed, long-term inhibitory effects would increase gastric function relative to the previous relaxed state. The polysynaptic projection of these neurons to the stomach implicates them in the autonomic regulation of gastric function, and suggests a direct link, therefore, between the rapid modulatory actions of glucocorticoids and eCBs and feeding/digestive behaviors.

## Conflict of Interest Statement

The authors declare that the research was conducted in the absence of any commercial or financial relationships that could be construed as a potential conflict of interest.
